# Intrathoracic drainage of a perforated prepyloric gastric ulcer with a type II paraoesophageal hernia

**DOI:** 10.1186/1749-7922-3-34

**Published:** 2008-12-08

**Authors:** Robert A Pol, Hiske W Wiersma, Bas JGL Zonneveld, Marinus Eeftinck Schattenkerk

**Affiliations:** 1 Department of surgery, Deventer hospital, PO Box 5001, 7416 SE, Deventer, The Netherlands; 2 Department of radiology, Deventer hospital, PO Box 5001, 7416 SE, Deventer, The Netherlands

## Abstract

**Background:**

With an incidence of less than 5%, type II paraesophageal hernias are one of the less common types of hiatal hernias. We report a case of a perforated prepyloric gastric ulcer which, due to a type II hiatus hernia, drained into the mediastinum.

**Case presentation:**

A 61-year old Caucasian man presented with acute abdominal pain. On a conventional x-ray of the chest a large mediastinal air-fluid collection and free intra-abdominal air was seen. Additional computed tomography revealed a large intra-thoracic air-fluid collection with a type II paraesophageal hernia. An emergency upper midline laparotomy was performed and a perforated pre-pyloric gastric ulcer was treated with an omental patch repair. The patient fully recovered after 10 days and continues to do well.

**Conclusion:**

Type II paraesophageal hernia is an uncommon diagnosis. The main risk is gastric volvulus and possible gastric torsion. Intrathoracic perforation of gastric ulcers due to a type II hiatus hernia is extremely rare and can be a diagnostic and treatment challenge.

## Case report

**Figure 1:**
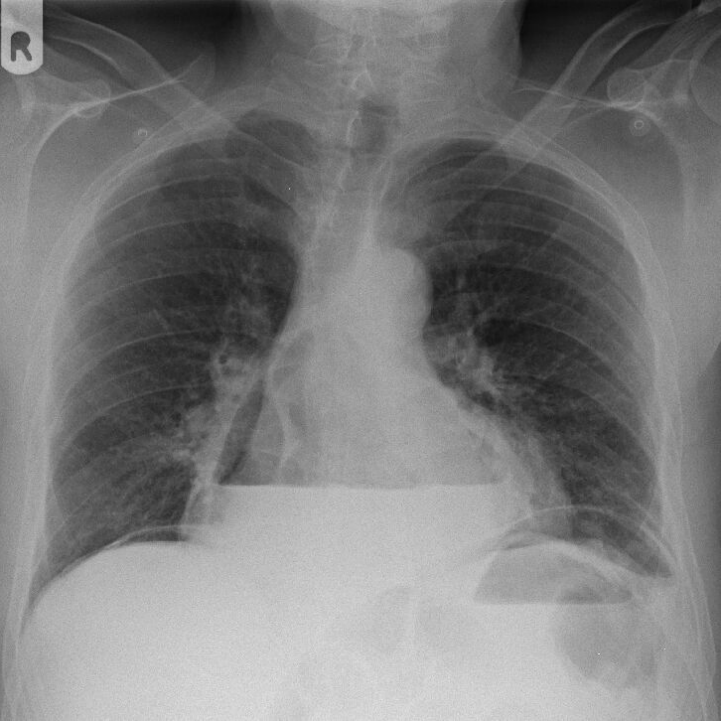
**Chest x-ray at the emergency department**. Posterior-anterior conventional radiograph of the chest with an intrathoracic air-fluid collection. Air-bubble in stomach. Free-intraperitoneal air inferior of both hemidiaphragms. Image suspect of stomach/bowel perforation and partial intrathoracic positioned stomach.

**Figure 2:**
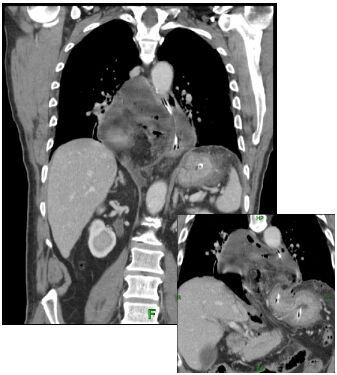
**CT-scan**. Coronal reconstruction CT-slice (3.7 mm). Intrathoracic mass consisting of mesenterial fat, free-intraperitoneal fluid and free-intraperitoneal air (1). On the left-side of this mass the esophagus is seen with a nasogastric tube (2) indicating a right-sided para-esophageal hernia with free intra peritoneal air and fluid. Intra-abdominal positioned stomach (3).

**Figure 3:**
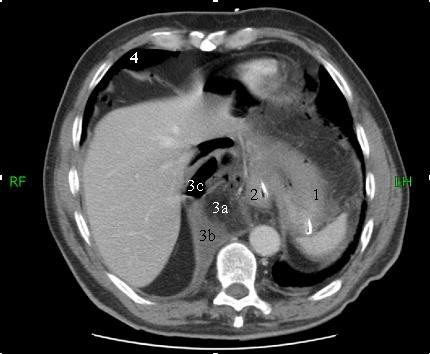
**CT-scan**. Axial 5 mm CT-slice after i.v. contrast admission. This slice shows an intra-abdominal situated stomach with nasogastric tube (1); esophagus with NGT (2); right-sided para-esophageal hernia with intraperitoneal fat (a), free-fluid (b) en free-air (c) (3); intra-peritoneal free-air (4).

A 61-year old Caucasian man with a previous medical history of a peptic ulcer, which was treated with a proton pomp inhibitor (PPI) and H. pylori eradication, presented with acute abdominal pain after several days of extensive vomiting and abdominal discomfort. On physical examination we saw a sick man with a grey appearance. His blood pressure was 190/120 mm Hg with a pulse rate of 100 beats/minute. Upon examination the abdomen was diffusely painful with guarding and rebound tenderness. Laboratory studies showed a normal renal function and electrolytes, a normal white blood cell count of 7,2 × 10^3 ^(4,0–10,0) and a slightly elevated c-reactive protein level of 25 mg/l (-10). On a conventional x-ray of the chest (Figure 1) a large mediastinal air-fluid collection and the suspicion of free intra-abdominal air, suggestive of a hiatal hernia and intra-abdominal perforation, was seen. In the differential diagnosis a Boerhaave syndrome was suspected. Additional computed tomography (CT) (Figure 2 and 3) revealed the aforementioned intra-abdominal free air and a large intra-thoracic air-fluid collection with a type II paraesophagal hernia. An emergency upper midline laparotomy was performed because of the septic profile and the suspicion of a perforated gastric ulcer. This revealed a perforated pre-pyloric gastric ulcer, which was treated with an omental patch repair. The greater omentum and fundus of the stomach were partially located in the paraesophageal hernia (analogous to the CT findings) and repositioned intraabdominaly. The intrathoracic collection was drained and extensively cleaned. The hiatal hernia was identified but left untreated, at the surgeon's discretion, because of the anticipated high risk of infection and abscess formation. Postoperatively broad-spectrum antibiotics (metronidazol/cefazoline) were continued for five days and a high dose of a proton-pump inhibitor was started. The patient fully recovered after 10 days and continues to do well.

## Discussion

Type II paraesophageal hernias are an uncommon diagnosis and occur in less than 5% of all hiatal hernias [1]. The etiology is still unclear but previous surgical interventions, such as antireflux procedures or partial gastrectomies, have been recognized as a known risk factor. Due to progressive enlargement of the phrenoesophageal membrane, the greater curvature of the stomach tends to roll up into the thorax. Eventually, the whole stomach herniates, forming an upside-down intrathoracic stomach [2].

Most patients with a type II hernia are asymptomatic or have mild gastroesophageal reflux disease (GERD) and are diagnosed during upper gastrointestinal endoscopy. The most important complications are gastric volvulus or bleeding from gastric ulcerations or erosions (Cameron lesions) [2,3]. The gold standard for gastric voluvus is open laparotomy with detorsion and anterior gastropexy, with or without a Nissen fundoplication [4].

Cameron lesions are linear gastric ulcers or erosions on the mucosal folds at the diaphragmatic impression in patients with a large hiatal herniam [3,5]. Unlike the pre-pyloric ulcer in this case, Cameron ulcers are located on the lesser curvature of the stomach. Treatment is primarily medical with acid suppressants and prokinetic agents [2,5].

To our knowledge only 5 case reports have been published reporting perforated gastric ulcers in combination with a paraesophageal hernia and just 2 cases reporting a perforated duodenal ulcer [6-12]. Normally, once a paraesophageal hernia is identified, it should be treated surgically with reduction of the herniated stomach with gastropexy to prevent reherniation and herniorraphy (or prosthetic mesh) of the diaphragma [13,14]. Debate exists whether or not an antireflux procedure is necessary. In this case no herniorraphy or mesh repair was carried out due to the anticipated high risk of infection and abscess formation. In the author's opinion risk of complications due to additional procedures should be avoided in view of the known high mortality of a perforated gastric ulcer in combination with a type II hiatus hernia.

## Consent

Written informed consent was obtained from the patient for publication of this case report and any accompanying images. A copy of the written consent is available for review by the Editor-in-Chief of this journal.

## Competing interest

The authors declare that they have no competing interests.

## Authors' contributions

RP drafted the manuscript as well as to the analysis of the literature. HW contributed to the conception and design of the manuscript, interpreted the radiology results, provided the figures and legends and revised the final manuscript. BZ and MES revised the final manuscript and provided important suggestions. All authors read and approved the final manuscript
